# Medullary adenocarcinoma of the ascending colon mimicking appendicitis after prior appendectomy

**DOI:** 10.1093/jscr/rjag949

**Published:** 2026-10-19

**Authors:** Ian L Cummings-Ruiz, Jose O Nieves-Munoz, Suheily Acevedo, Guillermo E Bolaños Avila

**Affiliations:** General Surgery Department, Hospital San Lucas, 909 Ave Tito Castro, Ponce, PR 00716, United States; General Surgery Department, Hospital San Lucas, 909 Ave Tito Castro, Ponce, PR 00716, United States; General Surgery Department, Hospital San Lucas, 909 Ave Tito Castro, Ponce, PR 00716, United States; General Surgery Department, Hospital San Lucas, 909 Ave Tito Castro, Ponce, PR 00716, United States

**Keywords:** medullary adenocarcinoma, ascending colon, appendicitis, right hemicolectomy, mismatch repair deficiency

## Abstract

Medullary adenocarcinoma is a rare colorectal carcinoma that may be difficult to recognize when inflammation dominates the presentation. A 65-year-old woman with a remote appendectomy presented with 1 month of postprandial abdominal pain, weight loss, and constipation. Repeated computed tomography demonstrated marked pericecal inflammation and a blind-ending structure interpreted as possible appendicitis despite her surgical history. Diagnostic laparoscopy identified an enlarged mesenteric lymph node, and excision unexpectedly showed metastatic medullary carcinoma of colonic origin with loss of MutL homolog 1 and PMS1 homolog 2 genes. Subsequent evaluation identified an ascending-colon primary. Right hemicolectomy confirmed a 1.5 cm poorly differentiated medullary adenocarcinoma, staged as pT2 pN1b, with negative margins. The patient remains clinically well during oncologic treatment. Persistent appendicitis-like inflammation, particularly when discordant with prior appendectomy, should prompt evaluation for cecal or ascending-colon malignancy.

## Introduction

Medullary adenocarcinoma is an uncommon colorectal carcinoma subtype with a predilection for the proximal colon, older patients, women, and mismatch repair-deficient tumors [1–3]. Its solid growth pattern and variable immunophenotype can complicate distinction from other poorly differentiated neoplasms [4]. We report an ascending-colon medullary adenocarcinoma that produced a persistent appendicitis-like inflammatory process in a patient with a remote appendectomy.

## Case report

A 65-year-old Hispanic woman presented with ~1 month of episodic postprandial abdominal pain radiating to the back, weight loss, and chronic constipation requiring daily laxatives. Her history included diabetes mellitus, hypertension, coronary artery disease, gastroesophageal reflux disease, asthma, appendectomy, and coronary artery bypass grafting. Her paternal grandmother and paternal uncle had colon cancer. She denied fever, vomiting, diarrhea, and hematochezia. She was well-appearing and had no peritoneal signs. Digital rectal examination showed no mass or blood.

Computed tomography demonstrated marked pericecal fat stranding and a thickened, ill-defined, blind-ending enteric structure interpreted as possible appendicitis, with inflammatory mass or adenopathy also considered. Carcinoembryonic antigen was 1.4 ng/ml, carbohydrate antigen 19–9 was 12 U/ml, and cancer antigen 125 was 8 U/ml. No colonoscopy or endoscopic biopsy was documented. Diagnostic laparoscopy showed dense adhesions involving the small bowel, cecum, and right abdominal wall. After adhesiolysis, an ~2 cm enlarged mesenteric lymph node was excised. Within several days, she returned with postoperative abdominal pain and nausea. Repeat computed tomography again showed the inflamed blind-ending pericecal structure. She improved with conservative management.

The excised specimen contained matted lymph nodes involved with metastatic medullary carcinoma interpreted as colonic in origin. Immunohistochemical markers of the tumor cells were strongly positive for cytokeratin AE1/AE3 and calretinin and negative for CK7, CK20, CDX2, and SATB2. Mismatch repair immunohistochemistry showed loss of MLH1 and PMS2 with retained MSH2 and MSH6. Magnetic resonance imaging subsequently showed a cecal mass with necrotic ileocolic adenopathy, three liver lesions, and a left lower-lobe pulmonary nodule considered suspicious for metastatic disease.

The patient underwent open right hemicolectomy with partial omentectomy. Operative findings included a large right-sided colonic mass with marked mesenteric desmoplasia, mesenteric lymphadenopathy, adjacent terminal ileal inflammation, right hepatic lobe nodularity, and a white omental plaque. An isoperistaltic side-to-side ileocolic anastomosis was created. No intraoperative complication was reported.

Resection pathology demonstrated a 1.5 × 1.5 × 1.2 cm poorly differentiated medullary adenocarcinoma of the ascending colon invading the muscularis propria. Small-vessel lymphovascular invasion, perineural invasion, and one tumor deposit were present. Two of 12 regional lymph nodes contained metastatic carcinoma. All margins were negative, the omentum was free of malignancy, and the tumor was classified as pT2 pN1b. Recovery was uncomplicated, and she was discharged on postoperative Day 4. Pulmonary nodules were clinically considered metastatic, and oncology initiated a planned 12-treatment course of pembrolizumab. At the most recent follow-up, during treatment 10, she reported no abdominal pain, tolerated oral intake, and was passing bowel movements. Distant disease had not been confirmed histologically.

## Discussion

This case is best organized around its diagnostic challenge. The patient presented with postprandial abdominal pain, weight loss, and constipation, while repeated imaging suggested an inflammatory right lower-quadrant process interpreted as appendicitis or an inflammatory mass. That interpretation was particularly discordant with her remote appendectomy. The case illustrates how an ascending-colon malignancy may produce appendicitis-like radiographic findings that initially obscure the underlying diagnosis.

Right-sided colon cancer may mimic acute appendicitis through local inflammation, luminal obstruction, or associated lymphadenopathy. A systematic review found right-sided colon cancer in ~1% of patients older than 40 years presenting with acute appendicitis, ˃10 times the risk in the general population [5]. Persistent pericecal inflammation in an older adult should therefore prompt direct evaluation of the cecum and ascending colon, particularly when imaging conflicts with the surgical history. Normal tumor markers should not exclude malignancy.

The diagnostic turning point was excision of the mesenteric lymph node rather than colonoscopy and endoscopic biopsy. Medullary adenocarcinoma predominantly affects the proximal colon and is strongly associated with mismatch repair deficiency [1–3]. Its solid architecture and variable immunophenotype can make distinction from other poorly differentiated neoplasms difficult, requiring correlation of morphology and immunohistochemistry [4]. Loss of MLH1 and PMS2 established mismatch repair deficiency but did not determine whether it was sporadic or hereditary. Guidance recommends BRAF V600E testing and/or MLH1 promoter methylation analysis when MLH1 is lost, followed by hereditary cancer evaluation when indicated [6]. This is especially relevant given the family history of colon cancer.

Definitive resection established a small pT2 primary despite nodal metastases, adverse histologic features, and one tumor deposit. The colectomy remained pT2 pN1b, while the pulmonary nodules were treated as clinically metastatic without tissue confirmation. The principal lesson is the importance of recognizing discordance between the clinical history and radiographic interpretation. Persistent appendicitis-like inflammation after appendectomy should broaden the differential diagnosis to include cecal and ascending-colon malignancy and prompt timely colonic evaluation or tissue diagnosis.

## Data Availability

The data supporting this case report are contained within the article. Additional clinical data are not publicly available to protect patient privacy.
